# BRD4 inhibition attenuates inflammatory response in microglia and facilitates recovery after spinal cord injury in rats

**DOI:** 10.1111/jcmm.14196

**Published:** 2019-02-26

**Authors:** Jianle Wang, Jiaoxiang Chen, Haiming Jin, Dongdong Lin, Yu Chen, Ximiao Chen, Ben Wang, Sunli Hu, Yan Wu, Yaosen Wu, Yifei Zhou, Naifeng Tian, Weiyang Gao, Xiangyang Wang, Xiaolei Zhang

**Affiliations:** ^1^ Department of Orthopaedics The Second Affiliated Hospital and Yuying Children’s Hospital of Wenzhou Medical University Wenzhou Zhejiang China; ^2^ Key Laboratory of Orthopaedics of Zhejiang Province Wenzhou Zhejiang China; ^3^ The Second School of Medicine Wenzhou Medical University Wenzhou Zhejiang China; ^4^ Department of Orthopaedics Affiliated Hospital of Guilin Medical College Guilin Guangxi China; ^5^ Department of Orthopaedics The Second Affiliated Hospital of Zhejiang University School of Medicine Hangzhou Zhejiang China; ^6^ Chinese Orthopaedic Regenerative Medicine Society Wenzhou Zhejiang China

**Keywords:** BRD4, inflammation, JQ1, microglia, spinal cord injury

## Abstract

The pathophysiology of spinal cord injury (SCI) involves primary injury and secondary injury. For the irreversibility of primary injury, therapies of SCI mainly focus on secondary injury, whereas inflammation is considered to be a major target for secondary injury; however the regulation of inflammation in SCI is unclear and targeted therapies are still lacking. In this study, we found that the expression of BRD4 was correlated with pro‐inflammatory cytokines after SCI in rats; in vitro study in microglia showed that BRD4 inhibition either by lentivirus or JQ1 may both suppress the MAPK and NF‐κB signalling pathways, which are the two major signalling pathways involved in inflammatory response in microglia. BRD4 inhibition by JQ1 not only blocked microglial M1 polarization, but also repressed the level of pro‐inflammatory cytokines in microglia in vitro and in vivo. Furthermore, BRD4 inhibition by JQ1 can improve functional recovery and structural disorder as well as reduce neuron loss in SCI rats. Overall, this study illustrates that microglial BRD4 level is increased after SCI and BRD4 inhibition is able to suppress M1 polarization and pro‐inflammatory cytokine production in microglia which ultimately promotes functional recovery after SCI.

## 1 INTRODUCTION

Traumatic spinal cord injury (SCI) is a devastating neurological condition which involves primary injury and secondary injury.^1^, ^2^, ^3^ Primary injury, resulting from the extraneous mechanical force impacting directly on the spinal cord, is characterized by focal cellular, vascular and blood‐spinal cord barrier injury; the primary injury of SCI is irreversible. Compared with primary injury in spinal cord, secondary injury is relatively remediable. The major events in the phase of secondary injury of SCI including oedema, ischaemia, inflammation, cell death and scar forming, among which inflammation is considered to be a major target for secondary injury.^2^, ^4^ Methylprednisolone is a corticosteroid thought to inhibit the inflammatory cascade contributing to secondary injury in SCI and it is the only drug that was recommended for SCI therapy; however later studies found methylprednisolone to be controversial for its efficacy and potential complications.^5^ Therefore targeted therapies for inflammation in the secondary injury of SCI are still lacking.

Microglia, the innate immune cells in the central nervous systems (CNS), are of vital importance for the repair of SCI.^6^ It is generally accepted that M1 polarization represents the pro‐inflammatory phenotypes and M2 polarization activates the anti‐inflammatory phenotypes in microglia.^7^ During development of SCI, M2 polarized microglia are able to remove apoptotic cells and inappropriate neural connections, whereas M1 polarized microglia in the injured spinal cord generate various pro‐inflammatory cytokines, such as TNF‐α, IL‐1β and IL‐6, which aggravate the secondary injury after SCI.^8^, ^9^

Inflammatory response of microglia is regulated by various pathways. NF‐κB is a well‐defined regulatory pathway of inflammation, it was reported to promote gene expression of pro‐inflammatory cytokines, many of which are up‐regulated in SCI^10^; suppression of NF‐κB signalling pathway limits pro‐inflammatory phenotypes and M1 polarization in microglia.^11^ Mitogen‐activated protein kinases (MAPKs) are considered as the key regulators of cellular processes such as inflammatory response and cellular stress.^12^, ^13^ Under pathological conditions, the phosphorylation of p38 mitogen‐activated protein kinase (MAPK), c‐Jun N‐terminal kinase (JNK) and extracellular signal regulated kinase (ERK) is markedly increased in microglia, which may lead to increased expression of pro‐inflammatory phenotypes such as TNF‐α, IL‐1β, IL‐6 and INOS.^13^, ^14^

Bromodomain‐containing protein 4 (BRD4) is a member of the Bromo and Extra‐Terminal (BET) family; it may bind to acetylated histones and transcription factors via bromodomains and regulate diverse pathophysiological activities including inflammation.^15^ BRD4 inhibition by JQ1 was demonstrated to attenuate LPS‐induced expression of pro‐inflammatory cytokines and suppressed inflammatory reactions in macrophages;^16^ studies also showed that JQ1 negatively modulates inflammation in rheumatoid arthritis and osteoarthritis indicating that suppression of BRD4 might be a potential therapeutic method against inflammatory response in SCI.^17^, ^18^

In the present study, we found that BRD4 level was correlated with pro‐inflammatory cytokines after SCI in rats. BRD4 inhibition either by lentivirus or JQ1 not only suppressed the MAPK signalling pathway but also inhibited the NF‐κB signalling pathway in microglia. Furthermore, JQ1 significantly attenuated M1 polarization and suppressed the expression of pro‐inflammatory cytokines in LPS‐treated microglia, which were similar to the results in vivo. Altogether, this study demonstrated that BRD4 inhibition can attenuate inflammatory response in microglia and BRD4 inhibition by JQ1 could be a potential novel therapy for SCI.

## 2 MATERIALS AND METHODS

### 2.1 Ethics statement

All surgical interventions, treatments and post‐operative animal care procedures were performed in strict accordance with the Animal Care and Use Committee of Wenzhou Medical University.

### 2.2 Reagent

(+)‐JQ1 was purchased from Meilunbio (Dalian, Shandong, China) and its purity was ≥98%. The lipopolysaccharide (LPS) was purchased from Sigma‐Aldrich (St Lousis, MO, USA). The primary antibodies for BRD4, IκBα, p‐p65, p65 and β‐actin were acquired from Abcam (Cambrige, UK). The p‐p38, p38, p‐JNK, JNK, p‐ERK and ERK antibodies were from CST (MA, USA). The IBA‐1 antibody was obtained from Genetex (Irvine, USA). 4', 6‐diamidino‐2‐phenylindole (DAPI) was purchased from Beyotime (Shanghai, China). Alexa‐Fluor‐488‐ and Alexa‐Fluor‐594‐tagged secondary antibodies were from Abcam. The reagents for cell culture were purchased from Gibco (Grand Island, NY, USA).

### 2.3 Cell culture, treatment and virus transfection

Highly aggressive proliferating immortalized microglia cells were purchased from BeNa Culture Collection (Beijing, China). The cells were cultured in Dulbecco's modified Eagle's medium (DMEM) containing 10% heat‐inactivated FBS and 100 units/ml penicillin and 0.1 mg/mL streptomycin in a humidified condition of 5% CO_2 _and 95% air at 37°C. HAPI microglia cells were treated with or without different concentrations of JQ1 for 2 hours and then LPS was added into the culture media at concentrations of 100 ng/mL or 1 μg/mL for different time. LV‐shBRD4 and LV‐Ctrl (MOI = 100) (Genechem, Shanghai, China) were added to HAPI cells for 12 hours and polybrene, that enhances the transfection efficiency of cells, was also added into the culture medium.

### 2.4 SCI animal model and treatment

Spinal cord injury protocols performed in female Sprague‐Dawley (SD) rats were described previously.^19^ Briefly, the rats were anaesthetized intraperitoneally with 10% (w/v) chloral hydrate (3.5 mL/kg). The operator performed a laminectomy located at the T9 vertebral section and removed the skin and muscles around the spinous processes to expose the vertebral column. The spinal cord was explicitly exposed and clamped using a vascular clip (10 newton force, Lingqiao Suture, Ningbo, China) for 60 seconds to establish a moderate crushing injury model. For the sham group, a T9 laminectomy was performed and the spinal cord was exposed for 60 seconds without compression injury. After surgery, rats from the JQ1 group and the SCI+JQ1 group were intraperitoneally injected with JQ1 (dissolved in DMSO solution) at a dose of 25 mg/kg bodyweight, whereas the other rats were intraperitoneally injected with DMSO solution at the same volume. We emptied the bladder twice daily after surgery until recovery of bladder function.

### 2.5 Western blot analysis

Spinal cord tissue from T7 to T10 was collected at a specific time after surgery. Briefly, the spinal cord tissue and HAPI cells were lysed using RIPA with phosphatase inhibitor and protease inhibitor cocktail and then the concentration of protein was measured using the BCA (Bicinchoninic Acid) protein assay (Beyotime, Shanghai, China); equivalent amounts of protein were separated using 8%‐12% SDS–PAGE gels and transferred to polyvinylidene fluoride membranes (Millipore, Massachusetts, USA). After blocking with 5% non‐fat milk for 2 hours, the primary antibodies were incubated: anti‐BRD4 (1:1000), p38 (1:1000), p‐p38 (1:1000), JNK (1:1000), p‐JNK (1:1000), ERK (1:1000), p‐ERK (1:1000), anti‐p65 (1:1000), anti‐p‐p65 (1:1000), anti‐IκBα (1:1000), anti‐INOS (1:1000), anti‐COX‐2 (1:1000), anti‐β‐actin（1:1000） followed by incubation with the respective secondary antibodies for 60 minutes. The resultant signals were detected using the ChemiDicTM XRS +Imaging System (Bio‐Rad) and the intensity of these bands was analysed using Image Lab 3.0 software (Bio‐Rad).

### 2.6 Histologic analysis and Immunofluorescence

Transverse and longitudinal sections (5 μm thick) were deparaffinized and rehydrated. Histopathological examination was performed with haematoxylin and eosin and Nissl staining as per the manufacturer's instructions. Bright‐field images were acquired by using light microscopy (Olympus, Tokyo, Japan). For immunofluorescence, HAPI microglia cells were fixed with 4% PFA for 15 minutes,and tissue or cell slices were blocked with 5% bovine serum albumin (BSA) for 30 minutes. Then, they were incubated with the following primary antibodies overnight: anti‐CD68 (1:100) and anti‐IBA‐1 (1:100). The next day, the following secondary antibodies were incubated for 1 hour: Alexa‐Fluor 488 goat anti‐rabbit and Alexa‐Fluor 594 goat anti‐mouse. Then, the slices were labelled with DAPI. All images were observed using a Nikon ECLIPSE Ti microscope (Nikon, Tokyo, Japan)**.**

### 2.7 RNA isolation and real‐time PCR

After treatment, the total RNA was extracted from the HAPI microglia cells in six‐well plates using TRIzol reagent (Invirogen, CA, USA). One microgram of total RNA was reverse‐transcribed to cDNA using 5× PrimeScript RT Master Mix (Takara, Japan) according to the manufacturer's instruction. For PCR amplification, 10 mL of reaction volume was used, including 5 mL of 2 × SYBR Premix Ex Taq mixture (Takara, Japan), 0.1 mmol/L of each primer, 1 mL of 2‐fold diluted cDNA and sterile distilled water. The reaction and detection were conducted in a light‐cycler (Roche, Mannheim, Germany). The ΔΔCt method was used to calculate the relative mRNA levels of each target gene. The primers for *Tnfα*, *Il1b* and *Il6* were listed as follows: *Tnfα *(F) 5ʹ‐AGCAAACCAAGCGGAGG‐3ʹ, (R) 5ʹ‐CAGCCTTGTCCCTTGAAGAGAAC‐3ʹ; *Il1b *(F) 5ʹ‐AGGAGAGACAAGCAACGACA‐3ʹ (R) 5ʹ‐CTTTTCCATCTTCTTCTTTGGGTAT‐3ʹ; *Il6* (F) 5ʹ‐AGGAGAGACAAGCAACGACA‐3ʹ(R) GGTCTGTTGTGGGTGGTATCCTC. The cycle threshold (Ct) values were collected and normalized to the level of the housekeeping gene *gapdh*. The 2^–ΔΔCt ^method was used to calculate the relative mRNA levels of each target gene.

### 2.8 ELISA analysis

Supernatants were collected from cell culture as well as tissue lysis and subsequently assayed for cytokine production. The cytokines TNF‐α, IL‐1β and IL‐6 were assessed using R&D Systems DuoSet ELISA (R&D Systems, Minneapolis, MN, USA) for rat TNF‐α, IL‐1β and IL‐6 (R&D Systems) following the manufacturer's protocol. The data were acquired by using Multiskan MK3 microplate reader (Thermo Fisher, Massachusetts, USA).

### 2.9 Locomotion recovery assessment

Locomotion recovery was assessed by using Basso‐Beattie‐Bresnahan (BBB) scores and footprint analysis at 1, 5, 7, 14 and 21 days. Rats were allowed to move freely in an open experimental field. Posterior limbs ability was assessed using the BBB scores ranging from 0 (no limbs movement or weight support) to 21 (normal locomotion). For footprint analysis, rat posterior and anterior limbs were dipped with red and blue dyes respectively. Evaluations were performed by five independent examiners who were blinded to the experimental conditions.

### 2.10 Statistical analysis

All data were expressed in means ± SEM format for at least three independent experiments. Statistical significance was analysed with one‐way analysis of variance（ANOVA）and Tukey's test using GraphPad Prism 6（La Jolla, CA, USA）for Windows. Differences were suggested to be statistically significant when *P* < 0.05.

## 3 RESULTS

### 3.1 Post‐traumatic inflammation activates BRD4 in microglia cells after SCI

**Figure 1:**
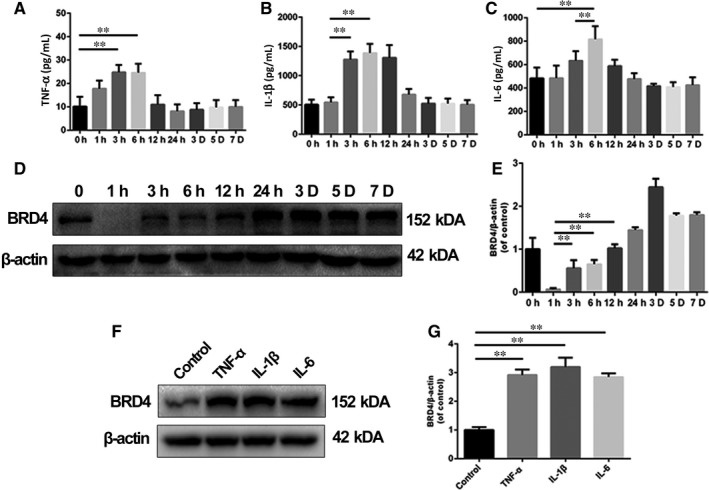
The level of pro‐inflammatory cytokines was correlated with the expression of Bromodomain‐containing protein 4 (BRD4). (A, B, C) The levels of TNF‐α, IL‐1β and IL‐6 were detected using ELISA in damaged spinal cord at different time points after spinal cord injury (SCI). (D, E) Representative images of western blots and quantification data of BRD4 expression at different time points after traumatic SCI. (F, G) Representative western blots and quantification analysis of BRD4 expression in HAPI microglia cells treated with TNF‐α (50 ng/mL), IL‐1β (50 ng/mL) and IL‐6 (25 ng/mL) for 24 h. All experiments were performed as mean ± SD of three times in duplicates. *P < 0.05, **P < 0.01

Pro‐inflammatory cytokines are reported to promote the level of BRD4 in different cells.^12^, ^17^ To evaluate the pro‐inflammatory microenvironment after SCI, we detected the levels of TNF‐α, IL‐β and IL‐6 in injured spinal cord. As shown in Figure 1A‐C, we found that the levels of TNF‐α, IL‐1β and IL‐6 increased and peaked at an early stage (earlier than 12 hours) after surgery indicating that microglial activation happens in the early stage of SCI. It is reported that BRD4 is implicated with pro‐inflammatory phenotypes in various cell types and microglia play a critical role in inflammatory response in the central nervous system.^17^, ^20^ To test BRD4 activation in microglia after SCI, we collected the T7‐T10 level around the lesion part of the spinal cord to detect the gross level of BRD4 in the spinal cord. Despite the sudden reduction at 1 hour after SCI, the level of BRD4 was increased after 1 hour of SCI, coinciding with the gradually elevated production of pro‐inflammatory cytokines at the early stage of SCI, which indicated that BRD4 might be enhanced in microglia and induced inflammatory response after SCI (Figure 1D,E). To confirm the effects of pro‐inflammatory cytokines on BRD4 in microglia, we detected the level of BRD4 in rat microglia cell line, highly aggressive proliferating immortalized (HAPI) cells, under the TNF‐α‐, IL‐1β‐ and IL‐6‐induced inflammatory conditions (Figure 1F,G). The results of the western blot assay showed that TNF‐α, IL‐1β and IL‐6 up‐regulated the BRD4 content in HAPI microglia cells. Altogether, our results suggest that post‐traumatic inflammation elevates the level of BRD4 in microglia after SCI.

### 3.2 Inhibition of BRD4 blocks LPS‐induced MAPK signalling pathway activation in microglia

Bromodomain‐containing protein 4 inhibition in chondrocytes was reported to reverse inflammatory response in the development of osteoarthritis, but the role of BRD4 in microglia activation is still unknown.^17^ Lipopolysaccharide (LPS) is a well‐recognized inflammatory response inducer in microglia, thus we used it to trigger inflammatory response in HAPI microglia cells.^21^, ^22^ Based on the activation of BRD4 after the SCI described above and previous studies about the pro‐inflammation property of BRD4, we decided to investigate the effects of BRD4 inhibition on inflammatory response after SCI.

**Figure 2:**
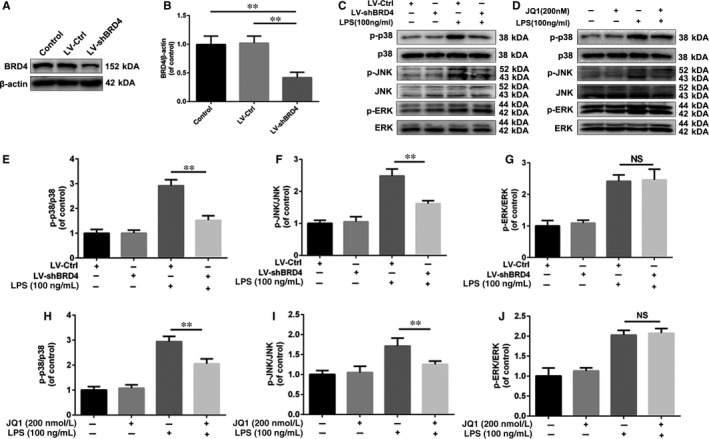
Bromodomain‐containing protein 4 knockdown and JQ1 block activation of MAPK signalling in microglia. (A, B) The BRD4 was silenced by lentivirus in HAPI microglia cells. (C‐F) Effects of BRD4 knockdown on phosphorylation of MAPKs (p38, JNK and ERK) in HAPI microglia cells. HAPI cells were administrated with LPS (100 ng/mL) for 1 h after pre‐treatment of JQ1 for 2 h. Then cells were collected and the cell lysate was subjected to western blots. (G‐J) Representative western blots and quantitative data for p‐p38, p‐38, p‐JNK, JNK, p‐ERK and ERK in HAPI microglia cells in the presence or absence of JQ1 or LPS. All experiments were performed as mean ± SD of three times in duplicates. *P < 0.05, **P < 0.01

As MAPKs, including p38, JNK and ERK, play essential roles in the regulation of inflammation, we explored whether BRD4 inhibition could affect the activation of the MAPK signalling pathway in LPS‐treated microglia. First, we knocked‐down BRD4 by using lentivirus in HAPI microglia cells (Figure 2A,B). As shown in Figure 2C‐F, LPS resulted in increased phosphorylation of p38, JNK and ERK, whereas BRD4 knockdown decreased LPS‐induced phosphorylation of p38 and JNK but not ERK. Also, similar results were observed in JQ1‐treated HAPI microglia cells under LPS‐induced inflammatory condition (Figure 2G‐J). These results indicate that BRD4 inhibition, either by lentivirus or JQ1, may suppress MAPK signalling pathway in LPS‐stimulated microglia.

### 3.3 Inhibition of BRD4 blocks LPS‐induced NF‐κB signalling pathway activation in microglia

**Figure 3:**
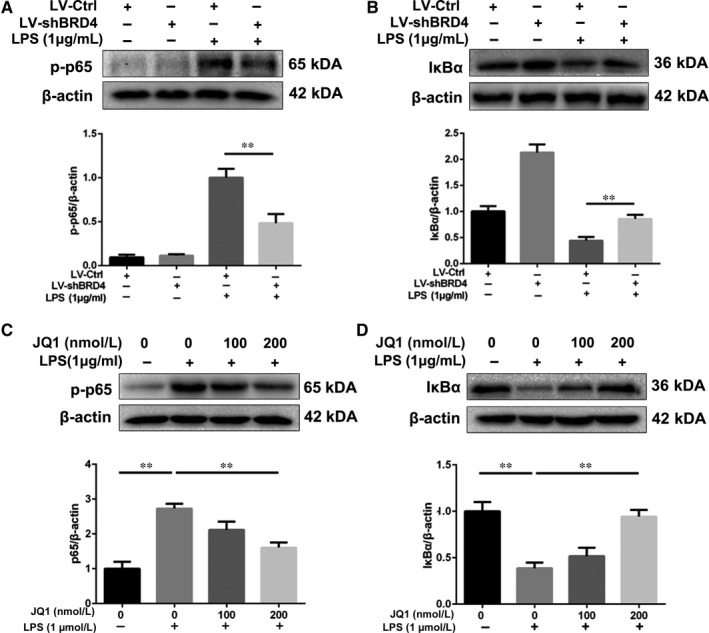
Bromodomain‐containing protein 4 knockdown and JQ1 alleviate the activation of NF‐κB signalling pathway in LPS‐treated microglia. (A, B) The effects of BRD4 knockdown on phosphorylation of p65 and the level of IκBα in LPS‐treated microglia. (C, D) Representative western blots and quantitative data for p‐p65 and IκBα in HAPI microglia cells with or without pre‐treatment of JQ1 for 2 h. All experiments were performed as mean ± SD of three times in duplicates. *P < 0.05, **P < 0.01

The NF‐κB signalling pathway is one of the most important regulating factors in microglial polarization, whose activation was accompanied with the phosphorylation of p65 and degradation of the inhibitor of NF‐κB (IκB).^23^, ^24^ To reveal the mechanism of BRD4 inhibition and JQ1 on inflammation, we detected the levels of two important mediators, p65 and IκBα, in the NF‐κB signalling pathway in LPS‐stimulated HAPI microglia cells. As shown in Figure 3A, LPS significantly promoted the phosphorylation of p65 in HAPI microglial cells, which was alleviated by BRD4 knockdown. However, the microglia without exposure to LPS displayed a low level of p‐p65. Interestingly, BRD4 knockdown also increased the level of IκBα in microglia treated with or without LPS (Figure 3B). As a BET antagonist, JQ1 inhibited the phosphorylation of p65 and promoted the level of IκBα in microglia in a dose‐dependent manner when HAPI microglial cells were treated with LPS (Figure 3C,D). These results suggest that BRD4 inhibition suppresses the NF‐κB signalling pathway in LPS‐stimulated microglia.

### 3.4 Inhibition of BRD4 by JQ1 prevents M1 polarization in LPS‐treated microglia

**Figure 4:**
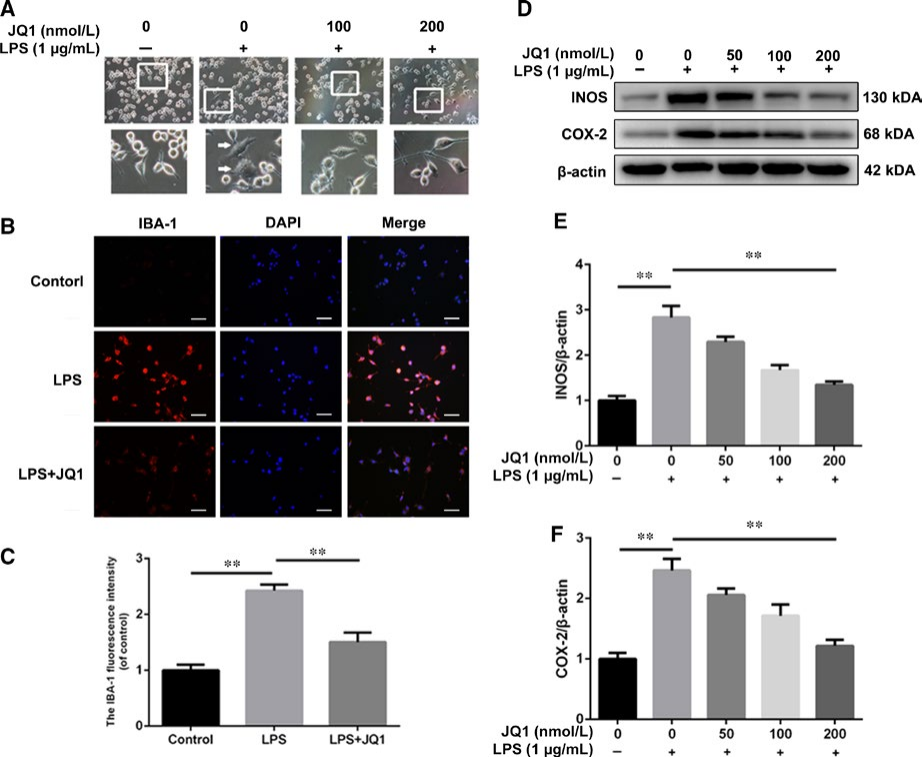
Bromodomain‐containing protein 4 inhibition by JQ1 attenuates the microglial M1 polarization. A, Morphological results of microglia treated with JQ1 or LPS. Scale bar: 100 μmol/L. LPS‐treated microglia displayed retracted branches, whereas pre‐treatment of JQ1 extended microglial branches under LPS‐induced inflammatory condition. HAPI microglia cells were treated with LPS (1 μg/mL) for 24 h. (B‐D) Immunofluorescence staining and quantitative data for IBA‐1 in microglia from different groups. Scale bar: 100 μmol/L. (E‐G) Representative western blots and quantitative data for INOS and COX‐2 expression in microglia from different groups. HAPI microglia cells were treated with or without LPS for 6 h. All experiments were performed as mean ± SD of three times in duplicates. *P < 0.05, **P < 0.01

M1 polarization represents pro‐inflammatory phenotypes in microglia leading to higher production of pro‐inflammatory cytokines.^25^ To explore the effects of BRD4 inhibition on polarization in microglia, we repressed BRD4 by using JQ1 in HAPI cells. As shown in Figure 4A, the results of the morphological study showed that HAPI cells transformed into amoeboid shapes after LPS stimulation, representing M1 polarization, which was prevented partly by treatment of JQ1. IBA‐1 was one classical M1 polarization marker for microglia. JQ1 compromised the fluorescence intensity of IBA‐1 in microglia treated with LPS (Figure 4B,C). Moreover, HAPI cells treated with increasing concentration of JQ1 displayed decreasing levels of INOS and COX‐2 under LPS‐induced inflammatory condition (Figure 4D,F).

**Figure 5:**
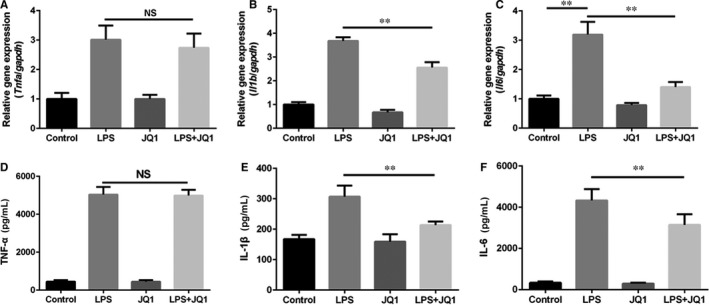
Bromodomain‐containing protein 4 inhibition by JQ1 suppresses the expression of pro‐inflammatory cytokines in microglia. Before exposure to LPS (1 μg/mL) for 6 h, HAPI microglia cells were treated with JQ1 (200 nmol/L) for 2 h. (A, B, C) Real‐time PCR assay of *Tnfa*, *Il1b* and *Il6* mRNA in HAPI microglia cells from each group as treated above. (D, E, F) ELISA measurements of TNF‐α, IL‐1β and IL‐6 from HAPI microglia cells in different groups. All experiments were performed as mean ± SD of three times in duplicates. *P < 0.05, **P < 0.01

Next, we detected the production of different pro‐inflammatory cytokines in JQ1‐treated microglia. Stimulated with LPS, HAPI microglia cells displayed higher mRNA levels of *Tnfa*, *Il1b* and *Il6* compared with the control group, whereas JQ1 decreased the mRNA content of *Il1b* and *Il6*, but not *Tnfa* (Figure 5A‐C). Similarly, as shown in Figure 5D‐F, JQ1 down‐regulated the levels of IL‐1β and IL‐6 but not the level of TNF‐α in the culture supernatants indicating that BRD4 inhibition of JQ1 is able to reduce secretion of IL‐1β and IL‐6 in LPS‐stimulated microglia. Altogether, our results show that inhibition of BRD4 by JQ1 negatively regulates the M1 polarization in LPS‐stimulated microglia.

### 3.5 BRD4 inhibition by JQ1 suppresses inflammatory response after SCI in rats

**Figure 6:**
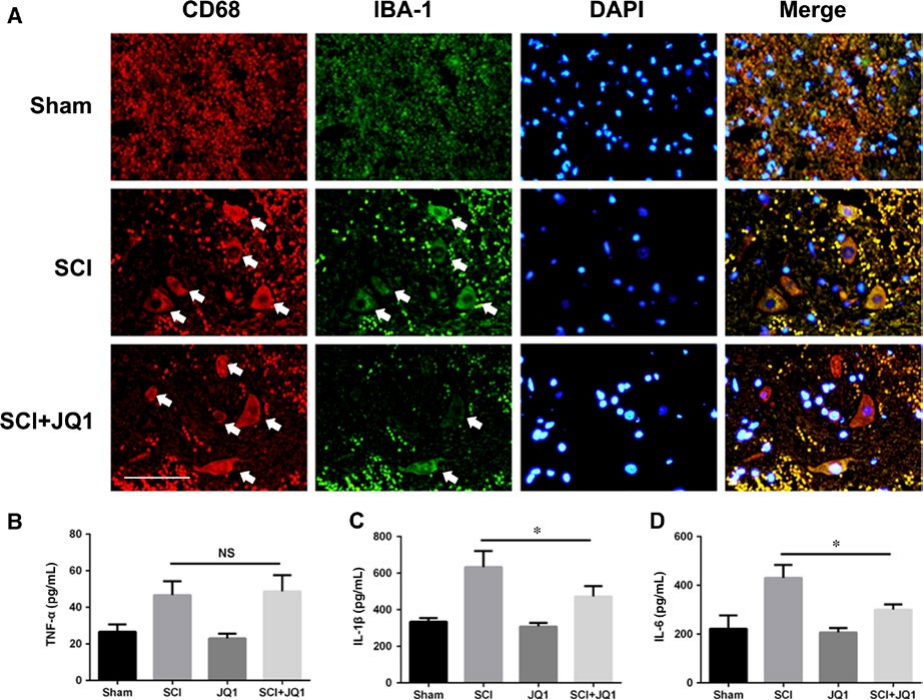
Bromodomain‐containing protein 4 inhibition by JQ1 suppresses inflammatory response after SCI. (A) Double immunofluorescence staining for CD68 (green) and IBA‐1 (red) positive microglia of sections from the tissue at 24 h after SCI. White arrows mark positive cells. Scale bar: 50 μmol/L. (B‐D) Quantification analysis of the levels of TNF‐α, IL‐1β and IL‐6 in spinal cord after 6 h after SCI. All experiments were performed as mean ± SD of three times in duplicates. *P < 0.05, **P < 0.01

Based on the anti‐inflammatory property of JQ1 in experiments in vitro, we examined the effects of JQ1 in rats after SCI. As shown in Figure 6A, the numbers of IBA‐1 and CD68 positive cells both increased in the SCI group, whereas administration of JQ1 reduced the level of these two M1 microglial markers in the lesion part of the spinal cord. These results suggest that inhibition of BRD4 by JQ1 blocks microglial M1 polarization in injured spinal cord in vivo. To test whether BRD4 inhibition by JQ1 is able to suppress levels of pro‐inflammatory cytokines in vivo, the levels of secretory TNF‐α, IL‐1β and IL‐6 were detected in the injured spinal cord at the early stage of SCI. As shown in Figure 6B‐D, the levels of these three cytokines increase after SCI, whereas the levels of IL‐1β and IL‐6 were reduced by JQ1; only TNF‐α was not affected. Our data shows that administration of JQ1 could reduce the secretion of pro‐inflammatory cytokines such as IL‐1β and IL‐6, but not TNF‐α, in impaired spinal cord.

### 3.6 BRD4 inhibition by JQ1 improves functional recovery and alleviates structural disorder as well as neuron loss after traumatic SCI in rats

**Figure 7:**
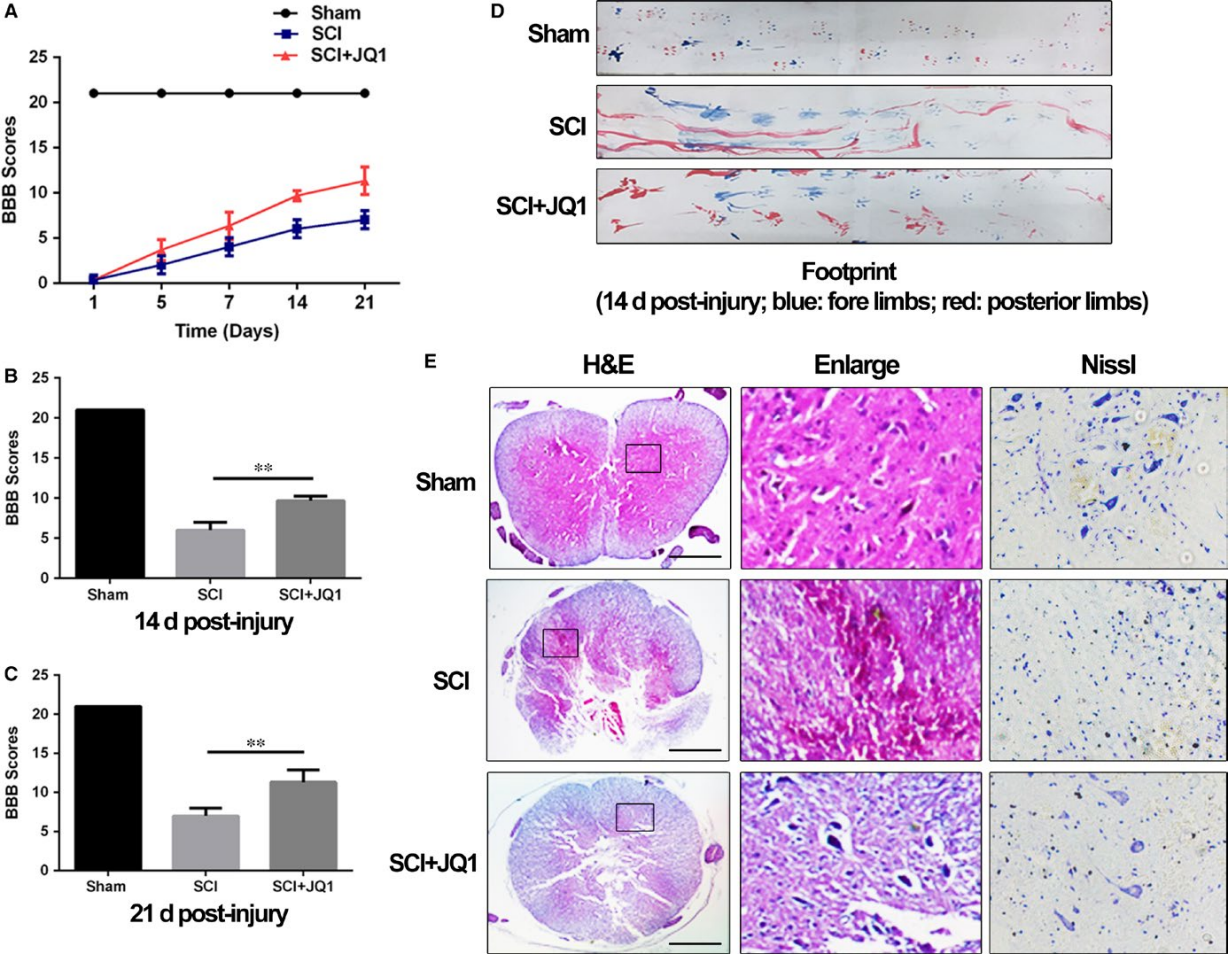
Bromodomain‐containing protein 4 inhibition by JQ1 improves functional recovery and attenuates structural disorder with less neuron loss in spinal cord after traumatic SCI. (A) The Basso, Beattie and Bresnahan (BBB) scores. (B, C) Quantification of BBB scores at 14 and 21 d. D, The footprint analysis of rats from Sham group, SCI group and SCI+JQ1 group. E, Representative images from haematoxylin and eosin and Nissl staining at 14 d after surgery. Scale bar: 1000 μmol/L. All experiments were performed as mean ± SD of three times in duplicates. *P < 0.05, **P < 0.01

Owing to the correlation between functional recovery and neuronal survival in SCI, we evaluated behavioural changes using BBB scores and footprint analysis. The results of BBB scores showed that SCI rat with no treatment displayed a lower functional recovery rate and maximum lower scores compared to those with JQ1 treatment after injury (Figure 7A‐C). Also, differences in tracks of posterior limbs were observed in footprint analysis. Compared with rats in the sham group that showed clear footprints, SCI rat with no treatment displayed extensive dragging of posterior limbs (red footprints), whereas SCI rats with JQ1 treatment showed fairly consistent posterior limbs tracks with little stumbling at 14 days after injury (Figure 7D). Moreover, the haematoxylin and eosin staining and Nissl staining were used to observe the morphology of spinal cord and neurons. As shown in Figure 7E, there was severer texture disorder with abnormal arrangement of nuclei and few neurons in the SCI group in contrast to rats from the sham group, whereas JQ1 improved the histological morphology and neurons survival. Thus, these findings suggest that BRD4 inhibition by JQ1 not only improves functional recovery but also reduces tissue disorder and neuron loss after traumatic SCI.

## 4 DISCUSSION

**Figure 8:**
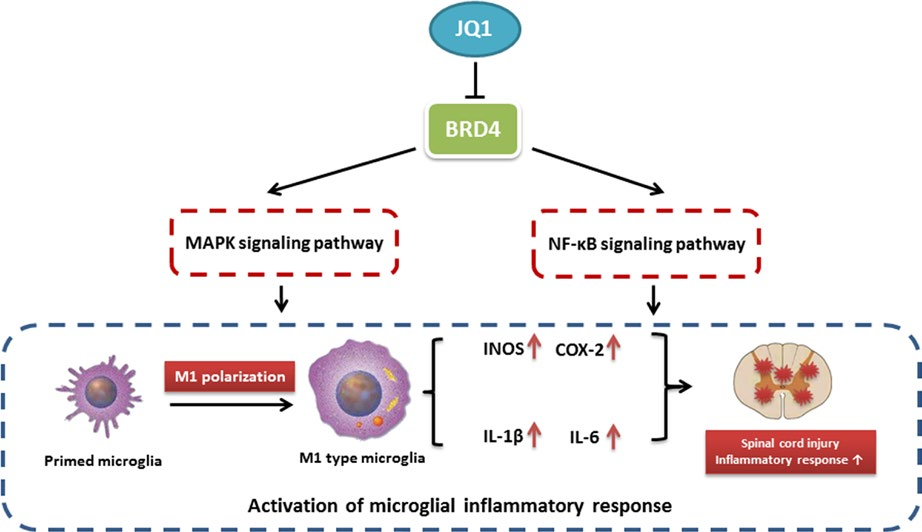
The schematic diagram depicting the molecular mechanism underlying the role of Bromodomain‐containing protein 4 in microglial inflammatory response after traumatic spinal cord injury (SCI)

This study provides evidence that BRD4 plays a critical role in modulating inflammatory response after SCI in rats. We found that (a) pro‐inflammatory cytokines promote the level of BRD4 in microglia after SCI; (b) BRD4 knockdown and JQ1 alleviate activation of signalling pathways of NF‐κB and MAPK in LPS‐treated microglia; (c) inhibition of BRD4 by JQ1 compromises the expression of pro‐inflammatory cytokines and M1 polarization in microglia; (d) administration of JQ1 reduces microglial M1 polarization and secretion of pro‐inflammatory cytokines in injured spinal cord; (e) BRD4 inhibition by JQ1 ameliorates secondary damage in spinal cord after SCI in rats. To the best of our knowledge, this is the first study exploring the role of BRD4 in inflammatory response regulation after SCI and describing a BRD4 inhibitor capable of suppressing inflammatory response via negatively modulating NF‐κB and MAPK signalling pathways (Figure 8).

BET family such as BRD4 are reported to activate many inflammation‐related genes,^26^ and inflammation blocks functional recovery and aggravates neuropathic paresthesia in damaged spinal cord.^27^, ^28^ Thus we explored the relationship between pro‐inflammatory cytokines and BRD4 in spinal cord after injury**. **In the present study, we found that the levels of different pro‐inflammatory cytokines such as TNF‐α, IL‐1β and IL‐6 were increased significantly at several hours after surgery, which might contribute to the increase of BRD4 level at the early stage of SCI. Correspondingly, the BRD4 levels were increased in TNF‐α, IL‐1β and IL‐6 treated microglia suggesting that microglial BRD4 was positively regulated by pro‐inflammatory cytokines after SCI in rats.

Bromodomain‐containing protein 4, amember of the BET proteins, has emerged as a crucial regulator in some inflammatory diseases.^12^, ^29^ JQ1, displacing BRD4 from acetylated lysines on chromatin, exerts prominent anti‐inflammation property in different diseases, but it has not been explored in traumatic SCI.^16^, ^29^ With treatment of JQ1, bone marrow‐derived macrophages display a lower expression level of key inflammation‐ and immunity‐related genes.^30^ Due to the critical role of BRD4 in inflammatory response in other cell types,^12^, ^29^ we explored the underlying mechanism of BRD4 inhibition on microglial activation in vitro. In this study we examined the effect of BRD4 inhibition on MAPKs and found that BRD4 suppression by lentivirus or JQ1 is able to reduce phosphorylation of p38 and JNK but not ERK, which was consistent with the results of previous study.^12^ In addition, BRD4 knockdown inhibits the NF‐κB signalling pathway by promoting the level of IκBα and preventing phosphorylation of p65 in IL‐1β‐treated chondrocytes.^17^ In line with these observations, we found that BRD4 knockdown and JQ1 both up‐regulated the level of IκBα and inhibited phosphorylation of p65, thus blocking the activation of the NF‐κB signalling pathway. All of these findings suggest that BRD4 inhibition depresses the NF‐κB and MAPK signalling pathways in microglia under the inflammatory condition.

Based on the property of BRD4 inhibition against MAPKs and NF‐κB in microglia, we detected the inflammatory response including pro‐inflammatory cytokine production and M1 polarization, in LPS‐treated microglia with pre‐treatment of JQ1. In this study, the LPS‐induced M1 polarization of microglia was reversed by JQ1, with less amoeboid‐shape microglia and lower levels of M1 microglial markers such as IBA‐1, INOS and COX‐2. Consistent with this observation, we found that JQ1 repressed the expression of IL‐1β and IL‐6 at transcription and translation levels in LPS‐treated microglia.

Another important evidence reported here is the capacity of BRD4 inhibition by JQ1 to attenuate the inflammatory response in vivo. Our data suggested that administration of JQ1 by injection intraperitoneally reduced the ratio of CD68 to IBA‐1 in the lesion spot of spinal cord. Besides, JQ1 decreased the levels of IL‐1β and IL‐6 in damaged spinal cord, but it did not affect the production of TNF‐α significantly in vivo, which coincided with the results of our experiments in vitro. These findings show that inhibition of BRD4 by JQ1 alleviates the inflammatory response in injured spinal cord. In addition, we found that JQ1 attenuates secondary damage after traumatic SCI with better functional recovery, improved structural disorder and less loss of neuron, indicating that JQ1 is an efficient molecule to alleviate traumatic SCI.

In our study, we found that JQ1 did not repress the expression of TNF‐α in vitro and vivo, which was similar to the previous study in which RNA sequencing of JQ1‐treated microglia under LPS‐induced inflammatory condition was performed.^30^ However, Belkina et al thought that JQ1 is a potential inhibitor of TNF‐α production in bone marrow‐derived macrophages.^16^ Therefore more investigations are required to explore the effect of BRD4 on TNF‐α expression. In addition, recent study showed that JQ1 isn‘t able to improve functional recovery in SCI mice, which might attribute to the difference of SCI model establishment, experimental animal species and JQ1 administration strategy.^31^

In conclusion, this study provided evidence for the role of BRD4 in inflammation regulation and showed that BRD4 inhibition was able to suppress inflammatory response in microglia via modulation of the NF‐κB and MAPK signalling pathways. Moreover, our results suggested that BRD4 inhibition by JQ1 may be a potential therapeutic method for attenuating inflammatory response to improve post‐traumatic recovery after SCI in rats.

## CONFLICT OF INTEREST

The authors declare no conflicts of interest.
